# The Incidence and Trends of Yellow Fever from 1990 to 2021 in Major Endemic Regions: A Systematic Analysis Based on the 2021 Global Burden of Disease Study

**DOI:** 10.3390/pathogens14060594

**Published:** 2025-06-16

**Authors:** Xinwei Wang, Bin Li, Baoren He, Xipeng Yan, Linbin Huang, Jinlian Li, Rongji Lai, Mingshuang Lai, He Xie, Qiuhong Mo, Limin Chen

**Affiliations:** 1 The Join-Laboratory of Transfusion-Transmitted Diseases Between Institute of Blood Transfusion, Chinese Academy of Medical Sciences and Nanning Blood Center, Nanning Blood Center, Nanning 530007, China; wangxinweivicky@163.com (X.W.); leo_li2323@163.com (B.L.); hebr2001@sina.com (B.H.); yanxipeng@163.com (X.Y.); huanglinbin2022@163.com (L.H.); jinlianli0902@163.com (J.L.); 18378805517@163.com (R.L.); lms99@student.pumc.edu.cn (M.L.); 2 The Hospital of Xidian Group, Xi’an 710003, China; ixiehe000@163.com; 3 Institute of Blood Transfusion, Chinese Academy of Medical Sciences and Peking Union Medical College, Chengdu 610052, China

**Keywords:** yellow fever, disease burden, incidence, trends, endemic regions

## Abstract

As a re-emerging disease, the worldwide burden and trends of yellow fever (YF) remain inadequately quantified. This study aims to assess the incidence of YF both globally and in major endemic regions from 1990 to 2021. Utilizing data from the Global Burden of Disease (GBD) database, we evaluated the burden of YF. We employed an age–period–cohort model to assess the influence of age, period, and cohort on the incidence of YF from 1992 to 2021. A secondary data analysis based on GBD database showed the following: in 2021, there were 86,509 incident cases of YF. Between 1990 and 2021, the number of incident cases decreased by 74.7%, while the age-standardized incidence rate (ASIR) declined at an EAPC of −4.76% (95% confidence interval: −5.10 to −4.42). In 2021, the highest ASIRs of YF were observed in Western Sub-Saharan Africa, Central Sub-Saharan Africa, and Eastern Sub-Saharan Africa. The analysis of age effects indicates that children aged 5–10 years old exhibit the highest incidence rate. Both period and cohort effects demonstrated a decline in morbidity risk. The decomposition analysis identified epidemiological changes as the primary factor contributing to the global reduction in the YF burden. Despite considerable reduction in incidence, YF remains a significant public health threat in Sub-Saharan Africa.

## 1. Introduction

Yellow fever (YF) is an acute viral hemorrhagic fever caused by the YF virus (YFV) infection. Before an effective vaccine was developed, YF was considered as one of the most feared and lethal zoonotic diseases, with a case fatality rate of up to 20–50% [1,2]. Recently, YF has re-emerged in tropical and subtropical regions of South America and Africa [1]. As of 2023, 34 countries in Africa and 13 countries in Central and South America are either endemic for YF or contain areas where the disease is endemic. The YF virus is transmitted among humans, mosquito vectors, and non-human primates (NHPs). The virus originated in Africa and was introduced to the Western Hemisphere and the Americas during the era of the transatlantic slave trade [3]. In subsequent centuries, outbreaks of YF were prevalent in tropical regions of the Americas, coastal cities in North America, and Europe. The introduction and widespread administration of vaccines in the mid-20th century resulted in a marked reduction in the disease’s incidence [4]. Nevertheless, there have been multiple YF outbreaks in the Americas and Africa in recent years [5,6], with exported cases to distant locations such as China [7,8]. This marked the first confirmed case in Asia, prompting concerns with the potential establishment of a YF transmission cycle in Asia. Factors conducive to YF epidemics in Asia include the presence of suitable vectors and the susceptibility of non-human primates [9]. Although indigenous YFV transmission has not yet been documented in Asia, the emergence of such a scenario remains possible [10].

YFV is a member of the family Flaviviridae, genus Flavivirus, and possesses a single-stranded, positive-sense RNA genome approximately 11 kilobases in length. This family includes other clinically relevant arboviruses such as dengue virus, Zika virus, and West Nile virus. In the majority of individuals, infection with the YFV may be asymptomatic or manifests with mild symptoms such as fever, headache, myalgia, and nausea. Approximately 20% of cases progress to more severe conditions, including jaundice, hepatic and renal failure, and hemorrhagic manifestations, with a case fatality rate ranging from 20% to 50% [11,12]. The YF vaccine is both safe and cost-effective, with a single dose conferring lifelong immunity; over 99% of vaccinated individuals develop protective antibodies within 30 days post-vaccination. Despite this, about half of the population in endemic regions remain unvaccinated [13]. In recent years there have been continued YF outbreaks, threatening millions residing in the tropical regions of South America and various parts of Sub-Saharan Africa [14,15], as well as to international travelers visiting these high-risk areas [16,17].

In response to the repeat outbreaks and high death rate of YF, the World Health Organization (WHO), in partnership with the United Nations Children’s Fund (UNICEF) and The Global Alliance for Vaccines and Immunization (Gavi), initiated an 11-year strategic plan in 2017, known as the Eliminate Yellow Fever Epidemics (EYE) strategy [18], with the objective of eradicating YF epidemics by 2026. This strategy encompasses three primary objectives: protecting at-risk populations, preventing international spread, and containing outbreaks rapidly. Despite the progress achieved under the EYE strategy, the prevention and control of YF continues to encounter numerous challenges, such as the inadequate and uneven global supply and distribution of vaccines. Furthermore, rising temperatures and extreme weather conditions have expanded the habitat range of Aedes aegypti mosquitoes, facilitating the virus’s spread to mid and high latitudes [19]. Additionally, insufficient surveillance and delayed outbreak responses have been predominantly observed in high-risk African nations. Although significant advancements have been made through the EYE strategy, complicated factors such as climate change and geopolitical conflicts continue to challenge the strategy’s long-term sustainability.

Factors such as global warming, accelerated urbanization, and intensified cross-border population movements are reshaping the epidemiological landscape of YF. Understanding the temporal trends and analyzing the unexplored demographic trend of YF epidemics is of urgent practical significance for optimizing the global prevention and control network. Previous investigations into YF have predominantly concentrated on the current epidemic state, neglecting to assess the individual contributions of age, period, and cohort effects to YF incidence. The Global Burden of Disease (GBD) study represents an international collaboration employing consistent methodologies and comprehensive population-level data to generate health indicators, thereby offering a unique opportunity to analyze disease trends on a global scale. In this study, we utilized data from GBD 2021 and age–period–cohort (APC) models to examine the global patterns, long-term trends, and regional variations in the incidence of YF infection from 1992 to 2021. The purpose of this current study is to provide epidemiological data support for policymakers and researchers with an aim to eliminate YF infections, particularly in high-risk areas where they are severely affected by this “neglected tropical disease” [20].

## 2. Materials and Methods

### 2.1. Data Sources

The GBD 2021 study offers a comprehensive evaluation encompassing 371 diseases and injuries, 288 causes of death, and 88 risk factors, stratified by age and gender across 204 countries and territories. In our research, we utilized the GBD Results Tool (available at: https://vizhub.healthdata.org/gbd-results/ (accessed on 7 February 2025)) to extract data on the number of YF cases and age-standardized rates from 1990 to 2021, organized into categories such as global, GBD region, country, gender, and age group. The GBD data primarily originated from the National Disease Surveillance System, Vital Registry System, Cause of Death Registry Report, and Population Survey data. The principal statistical methods and mathematical models employed in the estimation process include the Cause of Death Ensemble Model (CODEm), spatiotemporal Gaussian process regression (STGPR), and the Bayesian meta-regression tool DisMod-MR. These methodologies are applied to estimate prevalence, morbidity, mortality, years of life lost (YLLs), years lived with disability (YLDs), and disability-adjusted life years (DALYs) [21]. In countries with systematic underreporting, GBD estimates may be constrained by inadequate local data coverage. Rather than simply excluding the data, the research team weights, corrects, and predicts the data through a range of statistical methods. Countries with comprehensive vital registration systems are assigned higher data weights compared to those relying predominantly on survey data. For Sub-Saharan Africa, missing data are inferred from patterns observed in geographically proximate countries with similar socio-economic contexts. Additionally, a range of simulation outcomes can be produced using methods such as Monte Carlo simulations, or multiple imputation techniques can be employed to populate data across multiple simulated “samples”, ultimately synthesizing a more robust estimate. No ethical approval was required as the study used publicly available, de-identified data.

### 2.2. Age–Period–Cohort Analysis

The APC model decouples the age effect (physiological/social role change), period effect (external environmental shock), and cohort effect (intergenerational historical imprint) through mathematical modeling. Compared with analysis methods that only focus on a single time dimension (such as Joinpoint regression), APC can identify confounders more accurately and avoid misjudging cohort generational differences as age or period effects. The APC model is a widely recognized statistical tool employed in demographic, sociological, and epidemiological research. This model applies a log-linear Poisson framework to a Lexis diagram of observed rates, enabling the quantification of the additive effects attributable to age, period, and birth cohort. Our analysis primarily focused on evaluating parameters such as net drift and local drift, which are summarized as the annual percentage change in the overall population and age-specific ratios over time, respectively. The age effect elucidates factors such as the influence of population aging on morbidity and mortality rates. The period effect captures variations in disease and injury risk attributable to objective factors, while the cohort effect pertains to generational differences in exposure to disease risk factors. In this study, the incidence data for FY were categorized based on specific criteria. The age groups were segmented into 19 distinct categories, each spanning a 5-year interval, ranging from 0–4 years to 90–94 years. The temporal groups were divided into six successive 5-year periods, beginning from 1992 to 1996 and extending to 2017 to 2021. The birth cohort groups were delineated into 24 cohorts, covering the years 1898 to 2021, based on period–age analysis. The period from 2002 to 2006 and the birth cohort 1957–1961 were used as reference groups for calculating the relative risk (RR), with the period–age of 1986 serving as a benchmark. An RR value greater than 1 signifies an elevated risk of YF. The Wald chi-square test was employed to assess the significance of parameters and functions, with all statistical tests conducted as two-sided tests.

There are several limitations to APC analysis on GBD data. Firstly, the Global Burden of Disease (GBD) data are organized into 5-year intervals (e.g., 15–19 years), whereas the period data are collected annually. For the construction of an age–period–cohort (APC) model, it is imperative to aggregate the period data into 5-year intervals (e.g., 1990–1994). This aggregation may result in the loss of information or the smoothing of temporal effects, potentially obscuring annual variations in acute events, such as the 2016 Angola outbreak, and thereby affecting the model’s ability to capture short-term trends. Secondly, the APC model inherently assumes the independence of the age, period, and cohort effects. However, in practice, these effects may interact with each other. For instance, the influence of a vaccine rollout (a period effect) on children (an age effect) may differ based on the immunization history of the birth cohort, a complexity that GBD data may inadequately capture. Thirdly, the estimates produced by the GBD model may significantly diverge from actual monitoring data, and such systematic biases can be propagated into the APC model’s predictive outcomes. Furthermore, the APC model is subject to certain biases and risks of overinterpretation. Firstly, age, period, and cohort are intrinsically linearly related. The APC model assumes independence among these dimensions, potentially overlooking their interactions and influences. Consequently, the effects attributed to one dimension may be erroneously assigned to another. For instance, health impacts observed in a specific birth cohort following the introduction of certain vaccines might be misclassified as period effects, thereby neglecting their long-term cohort implications. Secondly, parameter collinearity can lead to logical inconsistencies in estimation results. Particularly in the presence of interactions, parameter estimates may deviate significantly from true values due to the competition for degrees of freedom. Lastly, the APC model is a descriptive statistical tool that does not account for unmeasured confounders, such as variations in educational attainment across cohorts.

### 2.3. Decomposition Analysis

A robust decomposition analysis is employed to discern the factors contributing to absolute changes in disease burden related to age. It allocates differences in incidence between two time points to changes in three independent factors: (a) Aging, which pertains to the alteration in the population’s age structure, specifically the shift towards a larger elderly demographic, commonly known as population aging; (b) Population, which involves changes in the overall population size; and (c) Epidemiological change, which encompasses the cumulative effect of all other factors beyond age structure and population size. The specific calculation method for this decomposition approach has been detailed in prior studies [22,23].

Between 1990 and 2021, we assessed the absolute and attributed proportions of the average annual variations in population growth, population aging, and epidemiological change across 21 GBD regions concerning the incidence of YF. The absolute contribution was the number of attributed incidence cases, while the relative contribution (“attributed proportion”) was estimated as the number of attributed incidence cases divided by the total number of deaths in 1990 × 100%. A positive contribution indicates an increase in total incidence, while a negative contribution indicates a decrease in total incidence.

### 2.4. Statistical Analysis

The prevalence of YF infection was quantified using the absolute number of YF cases, accompanied by a 95% uncertainty interval (UI), and the age-standardized incidence rate (ASIR), reported per 100,000 individuals. We documented the global incidence of YF infection and conducted an analysis of distribution patterns, concentrating on three principal YF-endemic regions: Western Sub-Saharan Africa, Central Sub-Saharan Africa, and Eastern Sub-Saharan Africa. Percentage Change (PC) was employed to assess trends in YF cases, while the EAPC was utilized to evaluate trends in ASIR. The EAPC was derived through linear regression analysis to assess temporal trends in disease burden. An increasing or decreasing trend in the age-standardized rate (ASR) was inferred if both the EAPC estimate and its 95% confidence intervals (CIs) were greater than zero or less than zero, respectively. Graphical visualization was conducted using the R software package (version 4.2.2) and JD_GBDR (version 2.35.1, Jingding Medical Technology Co., Ltd., Hefei, China). All *p*-values were two-sided, with statistical significance defined at a threshold of *p* < 0.05.

## 3. Results

### 3.1. Overview of the Global YF Incidence

**Figure 1:**
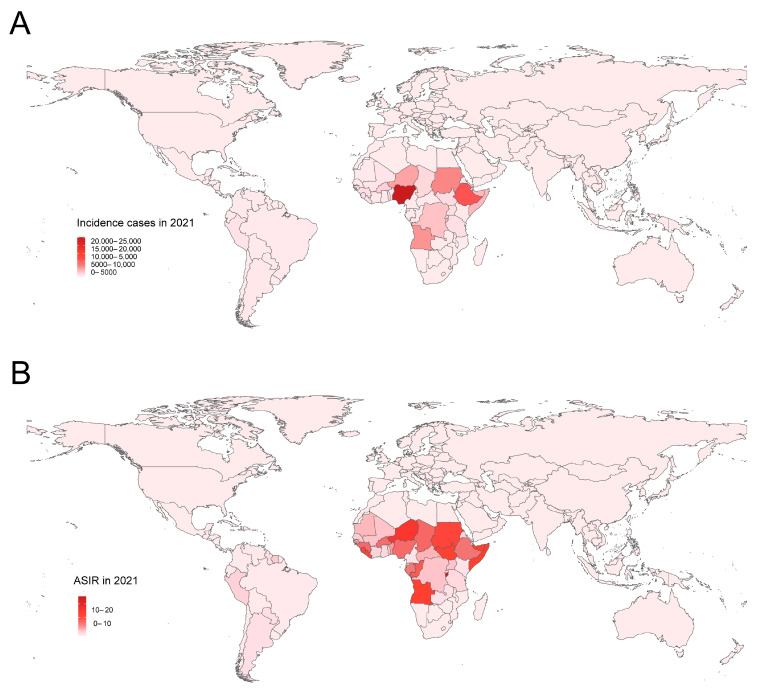
The global incidence burden of YF in 204 countries and territories in 2021. (**A**) The incidence cases; (**B**) ASIR. YF, yellow fever; ASIR, age-standardized incidence rate.

Globally, YF incident cases declined sharply between 1990 and 2021, from 341,942 (136,295 to 744,764) in 1990 to 86,509 (34,106 to 180,501) in 2021. This indicates a 74.7% decrease (95% UI: −70.85 to −78.02). Correspondingly, the ASIR decreased from 6.08 (2.42 to 13.21) to 1.15 (0.45 to 2.4) per 100,000 population, with an EAPC of −4.76 (95% CI: −5.10 to −4.42) (Table 1). YF is mainly found in Sub-Saharan Africa and Latin America (Figure 1). Compared with 1990, the incidence of YF has been decreasing in all nine endemic regions of the world, but the incidence of YF in Sub-Saharan Africa has always been dominant. The top three endemic regions were Western Sub-Saharan Africa, Central Sub-Saharan Africa, and Eastern Sub-Saharan Africa. In 2021, Burundi, Niger, Somalia, Angola, and South Sudan were the five countries with the highest incidence of YF, all in Sub-Saharan Africa (Table S1).

### 3.2. Time Trends on YF Incidence in Endemic Regions

**Figure 2:**
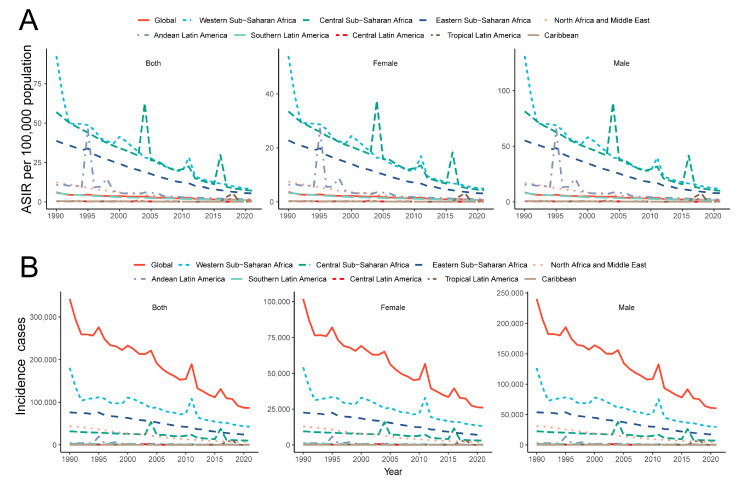
Temporal trend of incidence cases (**A**) and ASIR (**B**) of YF in major endemic regions from 1990 to 2021. YF, yellow fever; ASIR, age-standardized incidence rate.

As illustrated in Figure 2, the incidence of YF has consistently declined across all nine endemic regions. However, various outbreaks have occurred at different times in distinct areas. In the Andean region of Latin America, two outbreaks were recorded in 1995 and 1998, after which a reduced epidemic level was sustained. Central Sub-Saharan Africa experienced three outbreaks in 2004, 2011, and 2016. Additionally, an outbreak occurred in Tropical Latin America in 2016, predominantly affecting Brazil (Figure 2). Notably, the incidence rate is consistently higher in males than in females, with the male incidence rate being approximately twice that of females.

### 3.3. Age, Period and Cohort Effects on YF Incidence in Three Major Endemic Regions

**Figure 3:**
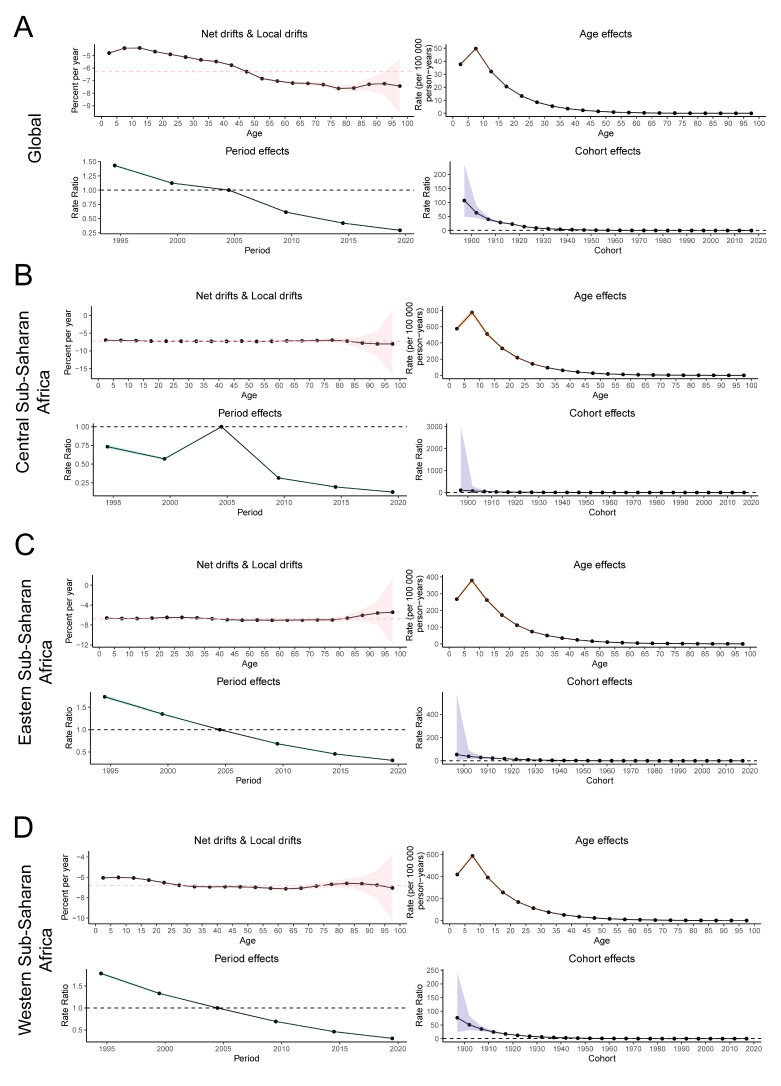
The net and local drifts and estimates of age, period and cohort effects on YF incidence rates in major endemic regions from 1992 to 2021. The local drifts illustrate the changes in YF incidence rates from 1992 to 2021 across 19 distinct age groups, ranging from 0–4 years to 90–94 years. The dots and shaded regions denote the annual percentage change in incidence rates (per year %) along with the associated 95% confidence intervals (CIs). The red dashed line represents the percentage change level of the 45–50 age group. The age effects are characterized by age-specific longitudinal ratios, which are adjusted for variations across different birth cohorts and account for period-specific biases. Period effects are depicted by the relative risk of yellow fever (YF) incidence across different time periods, calculated as the ratio of age-specific incidence from 1992–1996 to 2017–2021, with the baseline period designated as 2002–2006. Birth cohort effects are represented by cohort relative risks, calculated as the ratio of age-specific incidence between the 1897–1901 cohort and the 2017–2021 cohort, using the 1957–1961 cohort as the reference. The dots and shaded areas indicate the incidence or risk ratios and their respective 95% CIs. (**A**) global, (**B**) Western Sub-Saharan Africa, (**C**) Central Sub-Saharan Africa, (**D**) Eastern Sub-Saharan Africa. YF, yellow fever.

Figure 3 illustrates the local drift and the age, period, and cohort effects on the incidence of YF, expressed as the annual percentage change over time by age group, across global regions including Western, Central, and Eastern Sub-Saharan Africa. Between 1992 and 2021, the net drift in global YF incidence demonstrated a statistically significant decline of −6.28% (95% CI: −6.35 to −6.20) (Table S2). The local drift in YF incidence across all age groups worldwide was negative, indicating a consistent downward trend in incidence across all age groups, with the rate of decline accelerating with increasing age. Furthermore, the incidence of YF across the three major endemic regions exhibited similar patterns among different age groups (Figure 3).

A consistent age-effect pattern was identified both globally and across three high-risk regions, with children aged 5–10 years exhibiting the highest risk, which diminished with increasing age. From 1992 to 2021, the cyclical effect of YF demonstrated a decreased risk of onset globally and in the three major endemic regions. Nevertheless, Central Sub-Saharan Africa experienced an anomalous increase in risk between 2000 and 2005, potentially influenced by an outbreak. On a global scale, there is a general decline in risk within the birth risk cohort. The cohort effect exhibited the least reduction in Central Sub-Saharan Africa, while the most pronounced decrease was observed in Western Sub-Saharan Africa. Across all regions, the risk of morbidity reached its lowest level in individuals born in the most recent birth cohort compared to those born in the reference cohort (Figure 3).

### 3.4. Decomposition Analysis of YF Incidence Cases

**Figure 4:**
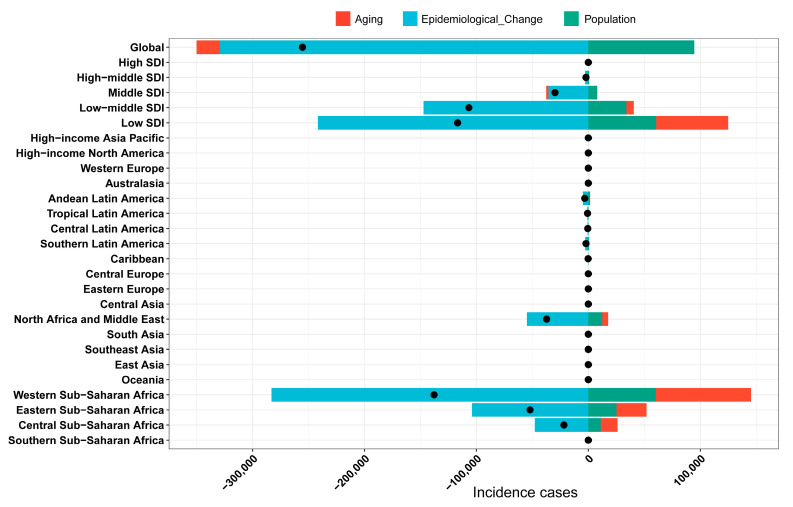
Decomposition analysis for YF incidence cases. YF, yellow fever. The black dots illustrate the comprehensive alterations in disease burden attributable to aging, epidemiological change, and population growth. For each component, an increase in the yellow fever (YF) disease burden is indicated by positive values, whereas a decrease is denoted by negative values.

The influence of factors such as aging, population growth, and epidemiological changes on the epidemiology of YF was assessed using a decomposition analysis of data spanning from 1990 to 2021 (Figure 4). Overall, the global incidence of YF exhibited a declining trend, with the most pronounced decrease observed in regions with a low socio-demographic index (SDI) and in Western Sub-Saharan Africa. On a global scale, aging, epidemiological changes, and population growth contributed 8.15%, 128.86%, and 37.01%, respectively, to the reduction in disease incidence (Table S2). Notably, aging remains a contributing factor to the YF disease burden in major endemic regions. Epidemiological changes have significantly contributed to the reduction in the global disease burden, particularly in Western Sub-Saharan Africa, with a contribution rate of 205.37%. The decomposition analysis underscores epidemiological changes as a primary driver of the global reduction in YF burden.

## 4. Discussion

In this study, we utilized the 2021 GBD data to evaluate the current status, long-term trends, and regional disparities in the global incidence of YF, with a particular focus on high-risk areas. To our knowledge, this represents the inaugural application of the APC model to analyze trends in YF prevalence. Overall, from 1990 to 2021, there was a substantial reduction in the burden of YF, evidenced by a decline in both the number of cases and the ASIR. Specifically, the global number of YF cases decreased from 341,942 in 1990 to 86,509 in 2021, with the ASIR declining from 6.08 per 100,000 to 1.15 per 100,000. Nonetheless, temporal trends in incidence exhibit significant variation across different regions and countries. Sub-Saharan Africa has consistently exhibited the highest incidence and the most rapid rate of decline.

This downward trend is closely associated with the intensification of global public health interventions, with the control of the yellow fever virus mainly relying on vaccination and mosquito vector control. Although YF cannot be eradicated due to its reservoir in non-human primates [24], vaccination can effectively eliminate the disease in human populations. The introduction of vaccines in the 1930s played a crucial role in the near eradication of YF in North America. During the period from the 1940s to the 1960s, West Africa gained significant benefits from mandatory mass vaccination campaigns, with most target countries achieving vaccination coverage exceeding 80%, which resulted in a prolonged decline in yellow fever incidence. However, between 1960 and 2000, vaccination efforts across Africa were constrained due to a discontinuity in vaccination campaigns. Consequently, the susceptible population increased as unvaccinated birth cohorts accumulated, leading to a resurgence of yellow fever on the continent. From 2006 to 2012, extensive vaccination campaigns were implemented in 11 West African countries and the Central African Republic as part of the yellow fever initiative. These campaigns enhanced vaccination coverage among the participating nations and are projected to reduce the overall burden in these 12 countries by 57% [25]. Initiated in 2017, the EYE campaign significantly augmented vaccination initiatives, resulting in a reduction in yellow fever (YF) incidence. In the same year, Hamlet [13] developed an interactive online tool, POLICI (Population-Level Immunization Coverage—Imperial) (accessible at: https://polici.shinyapps.io/yellow_fever_africa/ (accessed on 10 May 2025)), to facilitate the visualization and extraction of estimates regarding YF vaccination coverage across Africa. This tool integrates data from diverse sources, including historical mass vaccination campaigns, World Health Organization (WHO) reports, and academic literature, to compute and estimate vaccination coverage levels in Africa. The analysis of this tool’s data reveals that among the 36 African countries with epidemics, the average YF vaccine coverage increased from 15.5% in 1990 to 42.8% in 2017. Furthermore, WHO International Coordinating Group (ICG) data indicate that between 2018 and 2024, a total of 33,851,072 doses of the YF vaccine have been approved for deployment in Africa, thereby further enhancing vaccination coverage. Furthermore, most high-risk countries have integrated the YF vaccine into their routine immunization schedules [26]. Our findings demonstrate a significant inverse correlation between YF incidence and vaccination coverage, with incidence rates declining as coverage increased.

Early strategies for controlling yellow fever (YF) concentrated on the eradication of vectors in and around human settlements, with a primary focus on the principal vector, Aedes aegypti. The use of insecticides has been a prevalent method for managing mosquito populations, particularly during outbreaks, due to their ability to rapidly decrease mosquito density and disrupt virus transmission cycle. In 1905, a yellow fever outbreak in New Orleans highlighted the importance of mosquito vector control as an essential measure for epidemic prevention. Subsequently, in 1949, a comprehensive program was initiated across ten countries in the tropical regions of Central and South America with the objective of eliminating Aedes aegypti. This program achieved notable success, resulting in the eradication of mosquitoes from urban areas and effectively controlling the epidemic. The establishment of the African Centers for Disease Control and Prevention (CDC) in 2016 has enhanced the capacity for real-time sharing and monitoring of epidemiological data across Africa. Additionally, the EYE strategy has bolstered laboratory capacities for YF diagnostic testing, leading to improved surveillance, expedited diagnostic confirmation, and enhanced detection and response to outbreaks [27].

Nonetheless, the regional variability in the declining trend warrants careful consideration. Sub-Saharan Africa accounts for the majority of YF cases worldwide, with Western Sub-Saharan Africa, Central Sub-Saharan Africa, and Eastern Sub-Saharan Africa historically exhibiting the highest incidence rates. The substantial morbidity burden in Sub-Saharan Africa can be attributed to a multitude of contributing factors. Africa is widely regarded as the origin of YF [28,29], where the YFV has circulated in humans, NHPs, and mosquitoes for millennia. The continent’s extensive tropical rainforests and savannahs provide climatic conditions that are highly conducive to the propagation of YFV [30]. Concurrently, socio-economic factors significantly influence the persistent prevalence of YF. Inadequate medical resources, fragile healthcare systems, and the limited awareness of YF prevention and treatment exacerbate the YF epidemics [31]. Additionally, vector migration, climate change, and varying levels of YF immunity may contribute to YF outbreaks [32]. Climate change, characterized by increased precipitation and rising temperatures, has the potential to expand mosquito habitat ranges [33,34]. Furthermore, the transmission dynamics of forest-borne YF in Africa, characterized by the circulation of the virus between non-human primate hosts and wild Aedes mosquitoes [35], pose significant challenges for control efforts. This is in contrast to urban transmission, which primarily involves human-to-human transmission. This complexity renders it difficult to effectively disrupt the transmission chain solely through routine immunization programs.

YFV has seven main genotypes, five of which are distributed in Africa (Angola, East Africa, East/Central Africa, West Africa I, West Africa II) and two distributed in South America (South American genotypes I and II). Although different genotypes may be associated with different virulence to humans, the virulence phenotypes of YFV remain inadequately characterized. Research indicates that YFV maintains a strict geographically bound lineage dispersal pattern, with major genotypes circulating within strict regional confines [36]. The West African genotype is mainly found in West Africa, while the East and Central African genotypes are prevalent in their respective regions. In South America, genotype I is widespread in Brazil, Peru, and Colombia, whereas genotype II is rarer and localized. Our findings reveal occasional large outbreaks in Western and Central Sub-Saharan Africa, with a stable incidence in Eastern Sub-Saharan Africa. Further evidence is needed to determine if genetic differences among YF genotypes significantly influence outbreak distribution in Africa. Furthermore, certain genotypes of the yellow fever virus (YFV) may evolve into distinct genotypes as a result of adaptation to mosquito vectors. Some of these genotypes exhibit reduced virulence and are infrequently involved in yellow fever outbreaks. The resultant milder clinical manifestations often lead to missed diagnoses, thereby impacting the accuracy of incidence rate predictions.

Our study demonstrated that favorable age, period, and cohort effects were evident across all regions, contributing to a reduction in the incidence of YF. Research indicates that children aged 5–10 years are at the highest risk for YF, with the incidence rate peaking in this age group and subsequently declining with age. This pattern may be attributed to the underdeveloped immune systems of children or their increased frequency of outdoor exposure. The favorable period effect implies that prioritizing prevention and management strategies and implementing measures to control YF transmission are effective in regions with high YF prevalence. Maintaining funding for preventive campaigns in Central Africa—where forest transmission persists—is critical. Globally, and in all high-risk areas, the incidence of YF within birth cohorts has continued to decline, with cohorts born more recently exhibiting a significantly lower risk compared to earlier cohorts. This suggests that substantial progress has been achieved in all high-risk areas, notwithstanding regional variations in YF control, which underscores the need to protect routine immunization systems from disruptions. The risk of YF is anticipated to decrease further due to the incorporation of the YF vaccine into the routine immunization schedule in high-risk regions.

Despite advancements in YF prevention and control, several challenges persist. Firstly, although vaccine availability has significantly increased, vaccine coverage remains at an estimated 50% across the 42 countries and territories at risk in Africa and the Americas [37]. Vulnerable and at-risk populations, such as those working in agriculture, forestry, mining, and among migrant communities, remain unvaccinated, thereby heightening the risk of outbreaks and potential international spread. Secondly, although no indigenous cases have been documented in Asia, the ecological congruence between the distribution regions of the Aedes mosquito in Bangladesh and India closely parallels that of yellow fever-endemic areas, indicating a potential risk for transcontinental transmission. Furthermore, high-risk countries in Africa face challenges related to inadequate surveillance and delayed epidemic responses, compounded by the limitations of existing monitoring and evaluation frameworks, which hinder timely adjustments and corrections. Future success in combating YF will depend on multidisciplinary collaboration, equitable resource allocation, and adaptive strategies to address emerging risks.

Based on the aforementioned findings, we propose several recommendations to expedite the eradication of YF. Considering that Sub-Saharan Africa bears the highest burden of the disease, enhancing accessibility to vaccines in this region is imperative. This can be achieved by integrating large-scale vaccination campaigns with routine infant immunization programs, which has the potential to significantly reduce the disease burden and sustain low incidence levels through comprehensive coverage. For countries facing challenges in attaining high levels of expanded immunization coverage, an alternative strategy could involve conducting large-scale vaccination campaigns targeting children at regular intervals. Children aged 5 to 10 years are particularly vulnerable to yellow fever, making it essential to conduct targeted vaccination campaigns for this age group. Implementing age-specific booster doses for children aged 5–10 years old and utilizing schools and community centers to enhance vaccine accessibility is strongly suggested. The recurrent outbreaks in the African region are often linked to delayed monitoring, and the effects of climate change may further increase transmission risks. Therefore, establishing a regional yellow fever monitoring network to track climate data, predict changes in vector distribution, and proactively adjust key prevention and control measures is necessary.

Although this current study provides many useful epidemiological data for the management of YF, it suffers from several limitations. This study is based on available public health data and does not include field surveys or new model development. The estimation of YF infections in GBD study is contingent upon intricate statistical modeling and extrapolation methodologies. In areas with weak surveillance infrastructure, the accuracy of GBD estimates may be compromised due to incomplete reporting and diagnostic delays. The definition or measurement of a YF case may differ across geographical regions and temporal contexts, leading to potential variability in the accuracy and robustness of these estimates across different regional and health scenarios. Furthermore, mild or asymptomatic cases are often excluded from the surveillance system. Mild cases of YF are frequently underreported due to their non-specific symptoms and the limited surveillance or laboratory diagnostic capabilities in many vulnerable regions, potentially resulting in an underestimation of the YF burden. However, the burden estimation framework developed in this study offers significant new insights into the prevalence of yellow fever and the effects of vaccination efforts. This information is crucial for partners involved in the yellow fever initiative, as it enables them to assess the potential impact of future vaccination campaigns and to optimize the allocation of resources for yellow fever control effectively.

## 5. Conclusions

The worldwide incidence of YF is on a downward trend; however, notable regional and demographic disparities persist. Sub-Saharan Africa has consistently been characterized as a high-risk region. Despite significant reductions in morbidity due to vaccination efforts and surveillance systems, regional inequalities, ecological changes, and social vulnerabilities remain fundamental obstacles to the effective prevention and control of YF.

## Figures and Tables

**Table 1:** Incidence cases and age-standardized incidence rate of yellow fever in 1990 and 2021 and their temporal trends from 1990 to 2021.

	Incidence Cases			Age-Standardized Incidence Rate per 100,000		
Location	1990 No. (95% UI)	2021 No. (95% UI)	PC No. (95% CI)	1990 No. (95% UI)	2021 No. (95% UI)	EAPC No. (95% CI)
Global	341,942 (136,295, 744,764)	86,509 (34,106, 180,501)	−74.7 (−70.85, −78.02)	6.08 (2.42, 13.21)	1.15 (0.45, 2.4)	−4.76 (−5.10, −4.42)
Sex						
Male	240,110 (95,343, 520,309)	60,460 (23,848, 126,188)	−74.82 (−69.67, −78.69)	8.46 (3.38, 18.27)	1.57 (0.62, 3.28)	−4.81 (−5.15, −4.47)
Female	101,832 (38,880, 226,506)	26,048 (10,046, 56,233)	−74.42 (−69.96, −78.36)	3.65 (1.4, 8.12)	0.7 (0.27, 1.53)	−4.68 (−5.02, −4.34)
SDI						
High SDI	0 (0, 0)	0 (0, 0)		0 (0, 0)	0 (0, 0)	
High-middle SDI	2797 (733, 8971)	716 (173, 2008)	−74.41 (−49.14, −86.3)	0.26 (0.07, 0.86)	0.06 (0.01, 0.19)	−4.23 (−4.47, −3.99)
Middle SDI	32,091 (13,079, 72,973)	2356 (940, 5105)	−92.66 (−90.07, −94.27)	1.79 (0.73, 4.06)	0.1 (0.04, 0.22)	−6.61 (−8.01, −5.19)
Low-middle SDI	125,080 (44,978, 268,465)	18,320 (6525, 39,626)	−85.35 (−81.1, −88.45)	10.23 (3.71, 21.53)	0.91 (0.33, 1.99)	−6.67 (−7.17, −6.18)
Low SDI	181,882 (72,507, 405,491)	65,087 (25,975, 140,508)	−64.21 (−57.95, −69.19)	34.86 (13.89, 77.78)	5.35 (2.13, 11.61)	−5.91 (−6.34, −5.48)
GBD Regions						
Andean Latin America	4220 (1516, 9638)	1090 (356, 2541)	−74.17 (−66.98, −79.74)	10.78 (3.81, 24.72)	1.64 (0.54, 3.83)	−7.27 (−8.46, −6.07)
Australasia	0 (0, 0)	0 (0, 0)	0 (0, 0)	0 (0, 0)	0 (0, 0)	
Caribbean	200 (67, 548)	38 (12, 98)	−80.92 (−67.54, −88.39)	0.55 (0.19, 1.49)	0.08 (0.03, 0.21)	−5.81 (−6.18, −5.43)
Central Asia	0 (0, 0)	0 (0, 0)	0 (0, 0)	0 (0, 0)	0 (0, 0)	
Central Europe	0 (0, 0)	0 (0, 0)	0 (0, 0)	0 (0, 0)	0 (0, 0)	
Central Latin America	643 (235, 1403)	178 (66, 405)	−72.37 (−65.06, −78.26)	0.38 (0.14, 0.84)	0.07 (0.03, 0.16)	−5.36 (−6.50, −4.21)
Central Sub-Saharan Africa	32,028 (10,934, 77,516)	10,243 (3518, 24,801)	−68.02 (−57.73, −75.88)	56.96 (19.36, 139.34)	7.12 (2.44, 16.91)	−6.15 (−7.11, −5.19)
East Asia	0 (0, 0)	0 (0, 0)	0 (0, 0)	0 (0, 0)	0 (0, 0)	
Eastern Europe	0 (0, 0)	0 (0, 0)	0 (0, 0)	0 (0, 0)	0 (0, 0)	
Eastern Sub-Saharan Africa	76,461 (28,380, 174,014)	24,416 (8737, 57,219)	−68.07 (−57.11, −75.95)	38.77 (14.61, 87.97)	5.41 (1.94, 12.79)	−6.51 (−6.75, −6.27)
High-income Asia Pacific	0 (0, 0)	0 (0, 0)		0 (0, 0)	0 (0, 0)	
High-income North America	0 (0, 0)	0 (0, 0)		0 (0, 0)	0 (0, 0)	
North Africa and Middle East	43,918 (12,034, 129,407)	6659 (1684, 18,942)	−84.84 (−73.67, −91.25)	12.31 (3.48, 35.86)	1.04 (0.27, 2.94)	−8.16 (−8.43, −7.90)
Oceania	0 (0, 0)	0 (0, 0)	0 (0, 0)	0 (0, 0)	0 (0, 0)	
South Asia	0 (0, 0)	0 (0, 0)	0 (0, 0)	0 (0, 0)	0 (0, 0)	
Southeast Asia	0 (0, 0)	0 (0, 0)	0 (0, 0)	0 (0, 0)	0 (0, 0)	
Southern Latin America	2764 (714, 8858)	709 (168, 1998)	−74.33 (−48.54, −86.27)	5.54 (1.45, 17.61)	1.07 (0.25, 3.14)	−5.04 (−5.15, −4.93)
Southern Sub-Saharan Africa	0 (0, 0)	0 (0, 0)	0 (0, 0)	0 (0, 0)	0 (0, 0)	
Tropical Latin America	848 (343, 1936)	187 (75, 432)	−77.91 (−66.93, −85.69)	0.53 (0.22, 1.2)	0.09 (0.03, 0.2)	−2.99 (−6.29, 0.41)
Western Europe	0 (0, 0)	0 (0, 0)	0 (0, 0)	0 (0, 0)	0 (0, 0)	
Western Sub-Saharan Africa	180,859 (69,844, 399,593)	42988 (16,892, 92,256)	−76.23 (−71.73, −79.13)	92.63 (35.82, 205.27)	8.13 (3.24, 17.67)	−6.59 (−7.07, −6.11)

Abbreviations: CI, confidence interval; UI, uncertainty interval; EAPC, estimated annual percentage change; GBD, Global Burden of Disease; SDI, socio-demographic index.

## Data Availability

The datasets generated during the current study are available from the Global Health Data Exchange query tool (http://ghdx.healthdata.org/gbd-results-tool (accessed on 7 February 2025)).

## Supplementary Materials

The following supporting information can be downloaded at https://www.mdpi.com/article/10.3390/pathogens14060594/s1, Table S1: Incidence cases and age-standardized incidence rate of yellow fever in major endemic regions in 1990 and 2021 and their temporal trends from 1990 to 2021; Table S2: Age–Period–Cohort Model Analysis and drifts of yellow fever age-standardized incidence rate from 1992 to 2021.

## Abbreviations

The following abbreviations are used in this manuscript:

YF	Yellow Fever
YFV	Yellow Fever Virus
GBD	Global Burden of Disease
APC	Age–Period–Cohort
UI	Uncertainty Interval
CI	Confidence Interval
PC	Percentage Change
EAPC	Estimated Annual Percentage Change
ASIR	Age-Standardized Incidence Rate
