# Supplementary material for: The Incidence and Trends of Yellow Fever from 1990 to 2021 in Major Endemic Regions: A Systematic Analysis Based on the 2021 Global Burden of Disease Study
Source: Pathogens. 2025 Jun 16;14(6):594. doi: 10.3390/pathogens14060594 (PMC12195785; doi:10.3390/pathogens14060594)
Supplement: Supplementary file 1 [file pathogens-14-00594-s001.zip › pathogens-3592869-supplementary.pdf]

Supplementary materials

Supplement to: The Incidence and Trends of Yellow Fever from 1990 to 2021 in Major Endemic Regions: A Systematic Analysis Based on the 2021 Global Burden of Disease Study

Contents:

Supplementary Tables

Table S1. Incidence cases and age-standardized incidence rate of yellow fever in major endemic regions in 1990 and 2021 and their temporal trends from 1990 to 2021

Table S2. Age-period-cohort model analysis and drifts of yellow fever age-standardized incidence rate from 1992 to 2021.

Table S1. Incidence cases and age-standardized incidence rate of yellow fever in major endemic regions in 1990 and 2021 and their temporal trends from 1990 to 2021

Regions	Location	incidence cases			Age-standardized incidence rate per 100000		
		1990 No.(95%UI)	2021 No.(95% UI)	PC No.(95% UI)	1990 No.(95% UI)	2021 No.(95% UI)	EAPC No.(95% UI)
Central Sub-Saharan Africa	Angola	19438(5568.51222)	5717(1714.14391)	-70.59(-54.06.-80.84)	184.91(52.73.468.67)	16.73(5.09.43.66)	-7.18(-8.13,-6.21)
	Central African Republic	600(174.1644)	262(71.777)	-56.34(-31.5.-72.23)	21.38(6.31.60.28)	4.58(1.3.13.29)	-4.72(-5.37,-4.07)
	Congo	2719(791.8120)	671(198.1832)	-75.31(-63.33.-83.77)	108.39(32.61.310.83)	12.02(3.58.33.1)	-6.47(-7.62,-5.30)
	Democratic Republic of the Congo	7457(2083.19556)	3352(956.9079)	-55.04(-36.55.-66.41)	19.01(5.33.50.21)	3.58(1.04.9.79)	-5.16(-6.53,-3.78)
	Equatorial Guinea	703(187.1816)	73(18.194)	-89.55(-79.57.-94.03)	161.85(42.08.402.32)	4.52(1.2.12.49)	-11.90(-12.55,-11.24)
	Gabon	1111(345.2712)	167(50.408)	-84.96(-77.39.-89.8)	110.28(33.98.264.47)	8.91(2.67.21.41)	-7.92(-8.92,-6.90)
Eastern Sub-Saharan Africa	Burundi	6771(1948.18362)	3974(1071.11254)	-41.31(5.01.-65.04)	118.89(35.36.326.84)	29.07(8.06.82.75)	-4.88(-5.29,-4.46)
	Eritrea	687(188.1853)	163(47.448)	-76.22(-55.38.-	19.22(5.58.50.88)	2.36(0.7.6.47)	-6.55(-6.68,-

				86.18)			6.42)
	Ethiopia	43999(15716.104625)	10776(3451.26002)	-75.51(-63.11.-83.92)	84.29(30.92.193.58)	9.4(3.05.23.57)	-7.19(-7.56,-6.81)
	Kenya	2250(915.4994)	574(225.1274)	-74.5(-68.81.-79.62)	9.31(3.81.20.67)	1.06(0.42.2.37)	-6.58(-6.75,-6.41)
	Rwanda	1090(301.2964)	285(82.784)	-73.88(-51.53.-84.54)	14.59(4.24.38.92)	2.05(0.61.5.59)	-6.59(-6.86,-6.32)
	Somalia	4102(1079.11162)	3820(924.11699)	-6.86(88.63.-54)	49.63(13.83.133.47)	16.78(4.57.48.62)	-3.46(-3.50,-3.42)
	South Sudan	5378(1568.14265)	1627(442.4608)	-69.75(-46.13.-83.04)	89.23(25.42.233.78)	16.16(4.55.45.92)	-5.18(-5.59,-4.77)
	Uganda	7221(1950.20279)	1597(463.4482)	-77.88(-62.82.-86.67)	40.79(11.56.114.57)	3.51(1.01.9.22)	-7.94(-8.25,-7.63)
	United Republic of Tanzania	3911(1089.10628)	1305(370.3555)	-66.64(-38.34.-80.43)	14.65(4.26.39.13)	2.14(0.63.5.79)	-6.12(-6.29,-5.95)
	Zambia	998(277.2709)	274(77.751)	-72.5(-48.75.-83.84)	12.18(3.54.32.56)	1.34(0.4.3.65)	-7.29(-7.61,-6.97)
Western Sub-Saharan Africa	Benin	2009(564.5136)	879(273.2162)	-56.25(-33.49.-70.88)	40.07(11.61.95.78)	6.28(1.92.15.27)	-6.09(-6.69,-5.48)
	Burkina Faso	5436(1565.14097)	2324(663.6408)	-57.24(-39.71.-69.82)	55.25(16.28.142)	9.88(2.78.26.71)	-5.21(-6.84,-3.56)
	Cameroon	5673(1623.14855)	356(98.964)	-93.72(-92.1.-95.05)	53.12(14.84.135.95)	1.08(0.3.2.88)	-6.99(-8.26,-5.71)
	Chad	3758(1038.9444)	1931(503.4876)	-48.63(-17.61.-69.57)	61.2(17.29.160.23)	10.56(2.86.26.22)	-5.47(-5.97,-4.98)
	Côte d'Ivoire	2067(599.6299)	698(221.1901)	-66.22(-54.72.-74.49)	16.52(5.03.48.68)	2.43(0.76.6.6)	-6.14(-7.39,-4.88)
	Gambia	412(104.1019)	135(38.354)	-67.27(-41.91.-81.96)	41.36(10.81.104.95)	5.4(1.54.13.71)	-6.58(-6.87,-6.29)
	Ghana	2688(848.6373)	751(217.1797)	-72.08(-62.49.-78.68)	17.45(5.37.40.31)	2.11(0.62.5.15)	-6.82(-7.98,-5.65)
	Guinea	4383(1312.11685)	1455(397.3652)	-66.8(-52.96.-75.49)	72.11(21.82.185.67)	10.43(2.93.26.35)	-6.48(-7.86,-5.09)
	Guinea-Bissau	521(133.1535)	156(40.446)	-69.98(-41.15.-87.13)	49.62(13.55.136.16)	7.22(1.96.20.98)	-6.03(-6.12,-5.94)
	Liberia	2499(725.6246)	829(259.2168)	-66.81(-56.18.-74.83)	99.97(29.19.251.02)	14.63(4.55.37.23)	-7.59(-9.02,-6.14)
	Mali	2036(606.5558)	890(257.2533)	-56.27(-33.69.-72.56)	23.13(7.64.48)	3.57(1.06.10.02)	-5.95(-6.14,-5.76)
	Mauritania	728(202.1991)	200(55.549)	-72.49(-52.18.-84.04)	34.66(9.65.93.32)	4.37(1.25.11.96)	-6.58(-6.76,-6.41)
	Niger	6923(1702.19025)	4946(1233.14290)	-28.56(106.25.-70)	84.46(22.55.226.44)	18.89(5.16.53.15)	-4.84(-4.99,-4.68)
	Nigeria	135696(548	25429(98	-81.26(-	148(59.94	10.05(3.9	-6.98(-

	75.297396)	82.54182)	78.03.-83.84)	.323.22)	1.21.52)	7.38,-6.57)
Sao Tome and Principe	49(15.126)	10(3.27)	-80.13(-72.87.-85.43)	39.1(12.26.96.94)	4.34(1.35.11.7)	-7.17(-7.34,-7.00)
Senegal	1710(517.4482)	504(151.1292)	-70.53(-60.73.-78.1)	21.84(6.69.56.62)	3.05(0.92.7.85)	-7.31(-8.70,-5.89)
Sierra Leone	3850(1105.9789)	1348(381.3477)	-64.98(-52.28.-74.18)	91.51(26.35.231.08)	14.72(4.18.38.04)	-5.46(-6.77,-4.14)
Togo	414(110.1117)	145(42.392)	-65.05(-44.39.-77.97)	10.83(3.02.27.93)	1.66(0.48.4.39)	-5.60(-6.12,-5.08)

Abbreviations: CI, confidence interval; UI, uncertainty interval; PC, percentage change; EAPC, estimated annual percentage change.

Table S2. Age-period-cohort model analysis and drifts of yellow fever age-standardized incidence rate from 1992 to 2021.

	Global	Western Sub-Saharan Africa	Central Sub-Saharan Africa	Eastern Sub-Saharan Africa
Age				
< 5	37.72(37.06 to 38.4)	418.96(407.91 to 430.31)	575(547.49 to 603.9)	267.22(258.21 to 276.55)
5-9	49.75(48.9 to 50.61)	587.03(572.03 to 602.43)	775.45(739.64 to 812.99)	378.91(366.59 to 391.65)
10-14	32.16(31.62 to 32.71)	390.88(381.01 to 401.01)	509(485.93 to 533.17)	261.83(253.45 to 270.49)
15-19	20.64(20.3 to 20.98)	255.45(249.11 to 261.95)	331.59(316.92 to 346.94)	172.27(166.86 to 177.86)
20-24	13.31(13.09 to 13.52)	168.21(164.13 to 172.4)	219.14(209.71 to 228.99)	111.94(108.48 to 115.5)
25-29	8.54(8.41 to 8.68)	112.36(109.7 to 115.09)	141.88(135.97 to 148.05)	73.46(71.23 to 75.76)
30-34	5.51(5.43 to 5.6)	75.82(74.06 to 77.62)	94.48(90.67 to 98.45)	50.13(48.63 to 51.67)
35-39	3.61(3.57 to 3.66)	51.49(50.46 to 52.54)	62.22(60.01 to 64.52)	35.02(34.12 to 35.94)
40-44	2.34(2.31 to 2.37)	34.83(34.1 to 35.57)	41.49(39.98 to 43.05)	24.09(23.45 to 24.76)
45-49	1.49(1.47 to 1.51)	23.28(22.74 to 23.83)	26.97(26.03 to 27.96)	16.23(15.75 to 16.73)
50-54	0.94(0.93 to 0.96)	15.72(15.31 to 16.15)	17.54(16.75 to 18.37)	10.76(10.39 to 11.15)
55-59	0.59(0.58 to 0.6)	10.53(10.21 to 10.86)	11.53(10.91 to 12.19)	7.22(6.93 to 7.52)
60-64	0.36(0.35 to 0.37)	7.02(6.76 to 7.28)	7.34(6.86 to 7.84)	4.79(4.56 to 5.03)
65-69	0.22(0.22 to 0.23)	4.66(4.46 to 4.88)	4.79(4.41 to 5.2)	3.21(3.03 to 3.4)
70-74	0.14(0.13 to 0.14)	3.08(2.91 to 3.25)	3.13(2.82 to 3.47)	2.19(2.04 to 2.35)
75-79	0.08(0.08 to 0.08)	2.03(1.89 to 2.17)	2.03(1.77 to 2.32)	1.46(1.34 to 1.6)
80-84	0.04(0.04 to 0.05)	1.31(1.2 to 1.44)	1.33(1.1 to 1.6)	0.95(0.84 to 1.08)
85-90	0.02(0.02 to 0.03)	0.85(0.73 to 0.98)	0.87(0.64 to 1.17)	0.61(0.5 to 0.74)
90-94	0.01(0.01 to 0.02)	0.53(0.41 to 0.7)	0.54(0.3 to 0.99)	0.38(0.26 to 0.57)
95 +	0.01(0 to 0.01)	0.29(0.16 to 0.53)	0.29(0.06 to 1.3)	0.21(0.08 to 0.54)
Period				
1992-1996	1.43(1.42 to 1.45)	1.78(1.76 to 1.81)	0.73(0.71 to 0.76)	1.73(1.7 to 1.77)
1997-2001	1.12(1.12 to 1.13)	1.33(1.32 to 1.35)	0.57(0.56 to 0.58)	1.35(1.33 to 1.37)
2002-2006	1(1 to 1)	1(1 to 1)	1(1 to 1)	1(1 to 1)
2007-2011	0.61(0.61 to 0.62)	0.69(0.68 to 0.7)	0.31(0.31 to 0.32)	0.68(0.67 to 0.7)
2012-2016	0.42(0.42 to 0.43)	0.46(0.45 to 0.47)	0.19(0.19 to 0.2)	0.46(0.44 to 0.47)
2017-2021	0.29(0.29 to 0.3)	0.31(0.3 to 0.31)	0.13(0.12 to 0.13)	0.31(0.3 to 0.32)
Cohort				

1897-1901	106.89(48.67 to 234.76)	76.29(24.04 to 242.12)	102(3.48 to 2992.36)	54.34(5.23 to 564.94)
1902-1906	63.6(45.96 to 88.01)	50.74(31.35 to 82.14)	73.65(19.97 to 271.65)	38.45(16.63 to 88.88)
1907-1911	40.09(34.03 to 47.24)	35.18(27.57 to 44.89)	50.88(27.54 to 94)	28.84(19.61 to 42.4)
1912-1916	28.64(26.01 to 31.54)	24.62(21.26 to 28.49)	30.24(21.62 to 42.28)	23.03(18.7 to 28.35)
1917-1921	23.04(21.6 to 24.58)	17.23(15.6 to 19.03)	19.33(15.63 to 23.92)	18.35(16.09 to 20.91)
1922-1926	14.09(13.42 to 14.8)	12.15(11.27 to 13.09)	13.36(11.48 to 15.54)	12.5(11.33 to 13.79)
1927-1931	8.97(8.62 to 9.32)	8.89(8.38 to 9.43)	9.53(8.5 to 10.7)	8.83(8.16 to 9.54)
1932-1936	6.28(6.08 to 6.48)	6.32(6.02 to 6.63)	6.41(5.85 to 7.03)	6.19(5.81 to 6.6)
1937-1941	4.32(4.2 to 4.44)	4.39(4.22 to 4.58)	4.48(4.15 to 4.84)	4.31(4.08 to 4.54)
1942-1946	3.14(3.07 to 3.21)	3.01(2.91 to 3.11)	3.22(3.02 to 3.43)	2.96(2.83 to 3.1)
1947-1951	2(1.96 to 2.04)	2.08(2.01 to 2.14)	2.09(1.98 to 2.21)	2.06(1.98 to 2.14)
1952-1956	1.38(1.36 to 1.4)	1.44(1.4 to 1.48)	1.48(1.42 to 1.55)	1.45(1.4 to 1.5)
1957-1961	1(1 to 1)	1(1 to 1)	1(1 to 1)	1(1 to 1)
1962-1966	0.72(0.71 to 0.73)	0.7(0.69 to 0.72)	0.68(0.66 to 0.71)	0.69(0.67 to 0.71)
1967-1971	0.52(0.51 to 0.53)	0.49(0.48 to 0.5)	0.47(0.45 to 0.49)	0.48(0.47 to 0.5)
1972-1976	0.4(0.39 to 0.4)	0.34(0.33 to 0.35)	0.32(0.31 to 0.34)	0.33(0.32 to 0.34)
1977-1981	0.32(0.32 to 0.33)	0.24(0.23 to 0.24)	0.22(0.21 to 0.23)	0.24(0.24 to 0.25)
1982-1986	0.24(0.24 to 0.24)	0.17(0.16 to 0.17)	0.15(0.14 to 0.16)	0.17(0.17 to 0.18)
1987-1991	0.18(0.17 to 0.18)	0.12(0.12 to 0.12)	0.1(0.1 to 0.11)	0.12(0.12 to 0.13)
1992-1996	0.14(0.14 to 0.15)	0.09(0.08 to 0.09)	0.07(0.07 to 0.07)	0.09(0.09 to 0.09)
1997-2001	0.12(0.12 to 0.12)	0.06(0.06 to 0.07)	0.05(0.05 to 0.05)	0.06(0.06 to 0.06)
2002-2006	0.1(0.09 to 0.1)	0.05(0.05 to 0.05)	0.03(0.03 to 0.04)	0.04(0.04 to 0.05)
2007-2011	0.07(0.07 to 0.08)	0.03(0.03 to 0.04)	0.02(0.02 to 0.03)	0.03(0.03 to 0.03)
2012-2016	0.06(0.05 to 0.06)	0.02(0.02 to 0.03)	0.02(0.02 to 0.02)	0.02(0.02 to 0.02)
2017-2021	0.04(0.04 to 0.04)	0.02(0.02 to 0.02)	0.01(0.01 to 0.01)	0.02(0.02 to 0.02)
Local Drifts				
< 5	-4.79(-4.86 to -4.73)	-6.05(-6.13 to -5.96)	-6.99(-7.17 to -6.81)	-6.61(-6.73 to -6.49)
5-9	-4.41(-4.45 to -4.37)	-6.01(-6.07 to -5.95)	-7.07(-7.19 to -6.96)	-6.71(-6.78 to -6.63)
10-14	-4.39(-4.43 to -4.35)	-6.06(-6.11 to -6.01)	-7.13(-7.24 to -7.03)	-6.7(-6.77 to -6.64)
15-19	-4.68(-4.72 to -4.64)	-6.27(-6.33 to -6.21)	-7.25(-7.36 to -7.14)	-6.62(-6.69 to -6.55)
20-24	-4.9(-4.94 to -4.86)	-6.52(-6.58 to -6.46)	-7.27(-7.39 to -7.16)	-6.48(-6.56 to -6.4)
25-29	-5.12(-5.16 to -5.07)	-6.78(-6.85 to -6.71)	-7.29(-7.41 to -7.16)	-6.48(-6.57 to -6.4)
30-34	-5.34(-5.39 to -5.29)	-6.91(-6.99 to -6.84)	-7.29(-7.43 to -7.15)	-6.58(-6.68 to -6.49)
35-39	-5.47(-5.53 to -5.42)	-6.93(-7.02 to -6.85)	-7.29(-7.44 to -7.13)	-6.75(-6.85 to -6.64)
40-44	-5.77(-5.83 to -5.7)	-6.92(-7.02 to -6.83)	-7.3(-7.47 to -7.13)	-6.93(-7.05 to -6.81)
45-49	-6.28(-6.35 to -6.21)	-6.93(-7.04 to -6.83)	-7.25(-7.45 to -7.05)	-7.04(-7.18 to -6.9)
50-54	-6.84(-6.92 to -6.75)	-6.98(-7.1 to -6.86)	-7.34(-7.57 to -7.12)	-7.02(-7.18 to -6.86)
55-59	-7.03(-7.12 to -6.94)	-7.08(-7.21 to -6.94)	-7.3(-7.56 to -7.03)	-7.04(-7.23 to -6.86)
60-64	-7.19(-7.3 to -7.08)	-7.12(-7.29 to -6.96)	-7.18(-7.49 to -6.87)	-7.03(-7.24 to -6.81)
65-69	-7.22(-7.35 to -7.09)	-7.06(-7.26 to -6.87)	-7.16(-7.54 to -6.78)	-7.01(-7.26 to -6.75)
70-74	-7.31(-7.47 to -7.16)	-6.87(-7.1 to -6.63)	-7.1(-7.58 to -6.61)	-6.98(-7.29 to -6.66)
75-79	-7.62(-7.82 to -7.42)	-6.69(-7 to -6.38)	-6.97(-7.63 to -6.3)	-6.98(-7.39 to -6.57)
80-84	-7.59(-7.88 to -7.3)	-6.6(-7.04 to -6.15)	-7.26(-8.27 to -6.24)	-6.62(-7.25 to -5.99)
85-90	-7.29(-7.77 to -6.81)	-6.62(-7.35 to -5.89)	-7.79(-9.55 to -5.99)	-6.07(-7.19 to -4.93)
90-94	-7.24(-8.17 to -6.3)	-6.76(-8.15 to -5.36)	-8.05(-11.65 to -4.29)	-5.6(-7.99 to -3.16)
95 -100	-7.43(-9.6 to -5.2)	-7.04(-10.24 to -3.73)	-8.05(-16.9 to 1.73)	-5.44(-11.8 to 1.38)
Net drift				
	-6.28(-6.35 to -6.2)	-6.8(-6.91 to -6.69)	-7.28(-7.56 to -6.99)	-6.76(-6.94 to -6.57)
